# Modernizing Forensic Anthropology: A Data-driven Pipeline for Human Identification and Profiling

**DOI:** 10.1055/s-0046-1816537

**Published:** 2026-02-25

**Authors:** Samiya Riaz, Kawthar M. A. Bukhari, Sanjida Haque, Saira Atif, Muhammad Sohail Zafar, Sadia Syed

**Affiliations:** 1 Fundamental Dental and Medical Sciences, Kulliyyah of Dentistry, International Islamic University Malaysia, Malaysia; 2 Institute of Advanced Dental Sciences and Research, Lahore, Pakistan; 3 Department of Restorative Dental Science, College of Dentistry and Dental Hospital, Taibah University, Medina, Saudi Arabia; 4 University Dental Hospital, Taibah University, Medina, Saudi Arabia; 5 Center for Sustainability and Climate and Center of Excellence in Cybersecurity, Prince Sultan University, Riyadh, Kingdom of Saudi Arabia; 6 Department of Oral Biology, CMH Lahore Medical College and Institute of Dentistry, Lahore, Pakistan; 7 Department of Clinical Sciences, College of Dentistry, Ajman University, Ajman, United Arab Emirates; 8 Centre of Medical and Bio-allied Health Sciences Research, Ajman University, Ajman, United Arab Emirates; 9 School of Dentistry, Jordan University, Amman, Jordan; 10 Department of Forensic Medicine, Shifa College of Medicine, Islamabad, Pakistan

**Keywords:** forensic odontology, human identification, digital imaging, artificial intelligence, geometric morphometrics, CBCT

## Abstract

Forensic odontology has traditionally relied on dental morphology and odontometric measurements for identification and profiling purposes. Innovations in imaging technologies (high-resolution two-dimensional [2D] radiography, cone-beam computed tomography [CBCT], and intraoral three-dimensional [3D] scanning), geometric morphometrics analysis (GMA), and artificial intelligence (AI) have revolutionized the collection, analysis, and interpretation of dental data. Relevant literature was identified through searches in PubMed, Scopus, and Web of Science using the keywords forensic odontology, CBCT, GMA, AI segmentation, and human identification, focusing on English-language studies published between 2010 and 2025. This narrative review consolidates the existing evidence regarding (1) the enhancement of data acquisition and comparability through 2D and 3D imaging; (2) the quantification of dental sexual dimorphism by GMA and its application in machine learning (ML) classifiers; (3) recent advancements in sex-prediction models derived from tooth metrics and 3D shape data; and (4) the facilitation of dental model creation and identification workflows through AI-driven segmentation. The discussion encompasses practical benefits, existing limitations, validation requirements, and prospective directions for the adoption of this technique in forensic applications.

## Introduction

Dental structures are resilient and often endure conditions that obliterate soft tissues, rendering teeth essential for forensic identification and biological profiling (e.g., age and sex). Conventional odontometric and morphological methods employ calipers, radiography, and subjective evaluation. The advent of cost-effective 3D imaging (such as cone-beam computed tomography [CBCT] and intraoral scanners) and software applications for landmark identification and shape analysis has facilitated more accurate and consistent measurements. 1 Concurrently, AI, particularly convolutional neural networks (CNNs) and various machine learning (ML) algorithms, has demonstrated robust efficacy in automated tooth segmentation, landmark detection, and predictive modeling. 2 The integration of geometric morphometrics analysis (GMA) with ML provides a means for objective and high-precision sex estimation and expedites forensic processes. 3

Although previous reviews have discussed either AI-based or morphometric approaches in isolation, a comprehensive synthesis that combines digital imaging, GMA, and AI-driven automation within a unified forensic pipeline remains elusive. This review therefore distinguishes itself by bridging these domains to illustrate how modern computational and imaging technologies collectively modernize forensic identification and profiling. This study also identifies current challenges, validation gaps, and future research directions to guide the adoption of AI-assisted dental analyses in medico-legal settings.

## 2D Imaging in Forensic Odontology

Panoramic, periapical radiographs and digital images remain fundamental to medico-legal comparisons, as most antemortem dental records are 2D. They are fast, inexpensive, and familiar to practitioners, although issues such as magnification, distortion, and projection errors can reduce their precision. Standardized protocols and preprocessing techniques, including calibration and landmark correction, help mitigate these problems, allowing 2D odontometric indices to be applied effectively for sex estimation 4 and individual identification when 3D data are unavailable.

High-quality occlusal images provide additional value by capturing unique dental characteristics. Studies using digital photographs and stereomicroscopy have demonstrated the distinctiveness of occlusal groove patterns, confirming their strong individualizing potential for forensic comparison. 5 6 Research has also reported significant sex differences in maxillary first premolars and molars, with logistic regression models based on groove and occlusal parameters achieving 76.7% training accuracy and 70% testing accuracy in a Pakistani population. 7 These findings suggest that 2D occlusal images can support both identification and sex categorization, particularly when only antemortem photographs or casts are available, although validation on larger and more diverse datasets is required.

Beyond 2D approaches, sex prediction algorithms increasingly integrate odontometric measurements, geometric morphometric coordinates, and image-based characteristics. Modern computational pipelines can now generate 3D shape descriptors or convert geometric data into 2D feature maps for analysis using CNNs. The classifiers commonly employed in these studies include logistic regression, support vector machines, random forests, and deep learning architectures. Reported accuracies frequently exceed 90% in internal validation; however, this performance often declines when tested on external or population-diverse datasets. These findings emphasize the need for robust external validation and the development of population-specific models. 8 9

Recent studies have also explored hybrid methods that transform 3D tooth models into 2D representations suitable for CNN input, further illustrating the growing convergence between 2D and 3D descriptors in forensic odontology. 10 Collectively, these approaches highlight that although advanced machine learning methods show significant potential for sex estimation, their medico-legal reliability ultimately depends on model transparency, reproducibility, and validation across varied populations ( Table 1 ).

**Table 1:** Comparative summary of imaging modalities in forensic odontology

Imaging modality	Reported accuracy (typical range)	Major advantages	Key limitations	Approximate cost/accessibility
2D radiographs (panoramic, periapical)	70–85% (sex estimation, identification)	Widely available; inexpensive; compatible with most antemortem records	Projection errors; magnification and distortion; limited 3D detail	Low; routinely available in dental settings
Digital photographs	65–80% (groove pattern–based identification)	Simple acquisition; usable when radiographs unavailable	Lighting and angle variability; requires good-quality images	Very low; high accessibility
CBCT	85–95% (sex estimation, morphometrics)	Provides volumetric data; visualizes internal anatomy; useful for 3D superimposition	Higher radiation dose; equipment cost	Moderate to high; requires specialist setup
Intraoral scanners	80–95% (3D morphology, arch matching)	Non-invasive; high resolution; digital arch superimposition possible	Cost; learning curve; limited to surface features	High initial cost; increasingly available
Plaster dental casts/ resin casts	Percentage not available (supports identification validation)	Physical replicas; facilitates court presentation	Accuracy depends on scanner and printer calibration	Moderate

## 3D Imaging in Forensic Dentistry

3D imaging offers volumetric data and accurate spatial relationships, free from projection artifacts characteristic of 2D imaging. CBCT provides insights into internal anatomy and root morphology, which are beneficial for comparative studies and sex determination. 11 Intraoral and desktop surface scanners produce high-resolution crown morphology and occlusal anatomy suitable for 3D superimposition, 12 GMA. 13 Recent scoping investigations and pilot studies suggest that 3D superimposition techniques and surface-based superimpositions can provide reliable differentiation between individuals and improve pairing confidence where high-quality antemortem models are available. 14 3D data facilitate the extraction of intricate form features (curvature and landmark coordinates) which are essential for contemporary shape-based sex prediction methods. 15

## Geometric Morphometrics (GMA) Applied to Teeth and Sexual Dimorphism

GMA distinguishes between size and shape by employing landmark or semi-landmark coordinates to measure morphological variation. 16 In dental studies, GMA has been utilized on crowns, roots, and complete tooth surfaces to detect nuanced multidimensional variations across sexes that linear measurements may overlook. Numerous studies indicate that several dental elements—particularly canines and certain molars/premolars—exhibit significant sexual dimorphism in morphology and/or size, which GMA can quantify and utilize as characteristics for classification models. 13 The integration of GMA in neural networks and random forests has yielded encouraging accuracy in sex classification in preliminary datasets (often exceeding 80% for specific tooth types in rigorously controlled samples); however, performance is contingent upon demographic, sample size, and tooth selection.

**Fig. 1:**
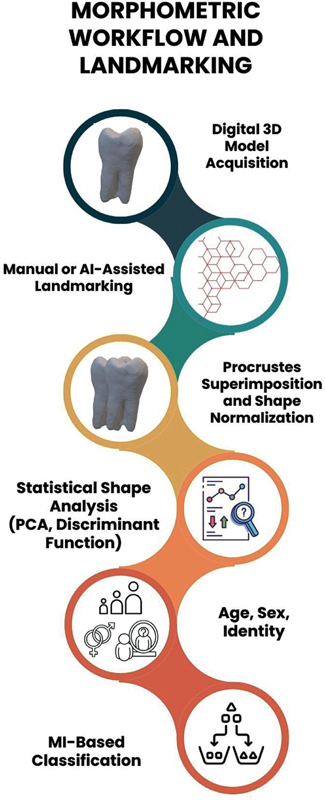
Schematic illustrating the geometric morphometric workflow, including 3D model acquisition, landmark placement, Procrustes alignment, shape quantification, and machine learning (ML)-based classification. PCA, principal component analysis.

Although GMA offers high precision in quantifying shape variation, its reproducibility depends on consistent landmark placements. Manual annotation often introduces inter- and intra-observer variability, which is influenced by operator experience and landmark definition. 11 12 13 Automated or semi-automated methods mitigate bias but may misplace landmarks on complex dental morphologies. Therefore, standardized landmarking protocols, observer calibration, and validated reference datasets are crucial to ensure accuracy and comparability across studies ( Fig. 1 ).

## Superimposition Techniques in Forensic Dentistry

Recent advancements in digital odontology have concentrated on employing 3D superimposition of the whole dental arches or partial dentition outlines to verify individuality and facilitate forensic human identification. Yu et al 14 established that 3D superimposition of human dental contours is an effective method for individual identification, emphasizing the significance of even minor morphological characteristics. Another study investigated 3D–3D teeth superimposition in an Eastern Chinese population, revealing remarkably reduced root mean square (RMS) distances in matches relative to mismatches, and attained 100% accuracy with a threshold of approximately 0.45 mm. 17 This highlights the possibility of full-arch superimposition in standard forensic procedure.

Prior research has also confirmed the efficacy of mandibular arch superimposition by CBCT and digital dental models, demonstrating consistent alignments that facilitate identification in partially damaged dental arches. 18 Another study indicated that six anterior teeth, particularly the labial surfaces, are adequate for reliable identification, especially in instances in which posterior teeth are missing or compromised. 19 Reesu et al investigated 2D–3D superimposition, and showed that the alignment of smiling images with 3D dental models markedly enhances reliability over visual assessment alone, thereby connecting clinical photographic documentation with forensic analysis. 20

Some studies have examined bitemark analysis with digital scans and 3D resin casts, validating the notion that dental morphology maintains originality regardless of whether it is captured via intraoral scanning or physical copies. 21 These studies collectively demonstrate that superimposition techniques applied to arches, tooth surfaces, 19 or biting patterns substantially augment the evidentiary significance of dentition in odontology investigations.

### AI for Dental Segmentation

One of the most labor-intensive aspects of forensic operations is the manual segmentation of teeth based on CBCT or scan data. 11 U-Net and nnU-Net are two examples of AI algorithms that facilitate automation and achieve high accuracy in regulated datasets. 22 Automation saves time, reduces the extent of operator variability, and standardizes inputs for subsequent analyses, such as GMA, 3D superimposition, and ML categorization. Moreover, rapid AI segmentation makes scalability and triage easier in mass-fatality scenarios.

## Integration of Imaging, GMA, and AI in Workflows

The acquisition of high-resolution 2D or 3D photographs of skeletal remains is often the first step in a viable forensic pipeline. These images are typically obtained through modalities such as computed tomography (CT) scans, light-structured scanning, or photogrammetry. Subsequently, the digital models are put through segmentation algorithms that are driven by AI. These algorithms segregate important anatomical features, such as the skull, pelvis, and even long bones, which are necessary for forensic examination. After the segmentation process is complete, GMA is performed to acquire landmark coordinates which capture shape variations that are absolutely necessary for biological profiling. 23

These coordinates function as quantitative inputs for ML classifiers, which are trained to predict biological characteristics such as sex, age at death, or individual identification. Algorithms for sex estimation may utilize pelvic or cranial morphology, whereas age estimation might be derived from tooth development, suture closure in the cranium, or patterns of bone density. Advanced models, such as ensemble learning and deep neural networks, may be trained using annotated datasets to attain high accuracy across varied populations. 24 25

Individual identification can also be achieved by comparing acquired morphometric data with established databases, employing methods such as form similarity scoring and probabilistic matching. To authenticate and illustrate the outcomes, 3D superimposition techniques can be employed, enabling researchers to superimpose query samples on reference models, thereby offering intuitive graphical evidence of matching or divergence. 14 15

To guarantee transparency and dependability, particularly in legal contexts, explainable AI methodologies are incorporated into the pipeline. These technologies provide interpretable outputs, including saliency maps, feature significance, and decision trees, rendering the system's conclusions comprehensible to non-expert stakeholders, such as judges or jurors. Moreover, uncertainty quantification techniques can effectively communicate confidence levels, thereby enhancing judicial defensibility. This comprehensive data-centric methodology integrates advanced technology with forensic protocols, facilitating more objective, accurate, and defensible forensic evaluations. 26

Various ML algorithms have been applied for biological profiling and dental identification, each differing in performance and interpretability. CNNs exhibit the highest internal accuracies, often exceeding 90%, owing to their capacity for automated feature extraction from CBCT or photographic data. 8 9 However, their reproducibility across populations declines in the absence of external validation. Support vector machines (SVMs) and random forest classifiers typically offer slightly lower accuracy (80–88%) but demonstrate greater stability and explainability in smaller or heterogeneous datasets. 9 24 Ensemble approaches that combine multiple models such as hybrid CNN–SVM or boosted decision trees achieve a balance between precision and generalizability, provided there are adequate training diversity and standardized preprocessing. Cross-population testing and transparent performance metrics (error rates, confidence intervals, and reproducibility scores) remain critical for model transferability and medico-legal reliability ( Table 2 ).

**Table 2:** Comparative summary of common machine learning algorithms used in forensic odontology and morphometric analysis

Algorithm	Typical accuracy range (%)	Key strengths	Limitations	Cross-population reproducibility	References
**Convolutional neural network (CNN)**	88–95	Automatically extracts complex image features; high internal validation accuracy	Requires large, population-specific datasets; limited interpretability	Often decreases on external datasets without retraining	8 9 24
**Support vector machine (SVM)**	80–88	Robust with smaller datasets; good for tabulated or geometric data	Needs feature engineering; moderate scalability	Moderate to high, depending on data normalization	9 24
**Random forest**	82–90	Handles mixed feature types; resistant to overfitting	Less effective for high-dimensional image data	High reproducibility if input features are standardized	24
**Ensemble/hybrid models**	85–93	Combines multiple algorithms (e.g., CNN + SVM) for improved balance between accuracy and generalization	Computationally intensive; complex optimization	Strong, if trained with diverse, multi-population datasets	24

## Limitations, Challenges, and Ethical Considerations

Despite major advances, several challenges must be resolved in forensic AI pipelines to achieve scientific rigor, ethical integrity, and legal admissibility.

### Population and Data Bias

ML models are often trained on limited or geographically constrained datasets, leading to reduced accuracy when applied to diverse populations. Population-specific baselines and inclusive datasets are essential for equitable medicolegal application. 27

### Annotation and Benchmark Variability

Manual landmark annotation remains prone to inter- and intra-observer variability, which affects the reliability of downstream models. 28 29 30 Although automated landmarking reduces inconsistency, it has its limitations. 29 The absence of universal benchmarks and standardized performance metrics (e.g., error rates, confidence intervals, and reproducibility parameters) further complicates the comparison and validation of forensic AI models. 31 The establishment of shared datasets and metrics will enhance methodological transparency and comparability.

### Data Privacy, Transparency, and Ethical Safeguards

Dental and craniofacial records represent sensitive biometric data that require stringent safeguards. Their use must comply with privacy laws, such as the general data protection regulation (GDPR), ensuring anonymization, restricted access, and informed consent. 32 Equally critical is algorithmic transparency; AI systems must produce interpretable outputs, such as saliency maps or feature importance—to support explainability and judicial comprehension. 33 Reporting uncertainty and performance variation across age or population groups strengthens admissibility and public trust. 34

### Handling Partial or Degraded Data

AI models often assume complete and high-quality inputs. Degraded or incomplete remains (fractured bones, missing landmarks, or distorted scans) can drastically reduce the performance of the model. 35 Therefore, robust imputation methods or adaptable algorithms are required for real-world forensic cases where data quality is variable. In addition, inconsistent imaging modalities, preprocessing, and segmentation approaches can introduce systematic discrepancies and degrade model performance. 36

## Legal and Regulatory Validation

Even scientifically sound models can be inadmissible in court without verifiable validation, reproducibility, and compliance with legal standards, such as Daubert's Rule and Frye Guidelines. 37 Maintaining a clear chain of custody, standardized imaging protocols, and traceable documentation is essential for ensuring legal defensibility.

## Conclusion

**Fig. 2:**
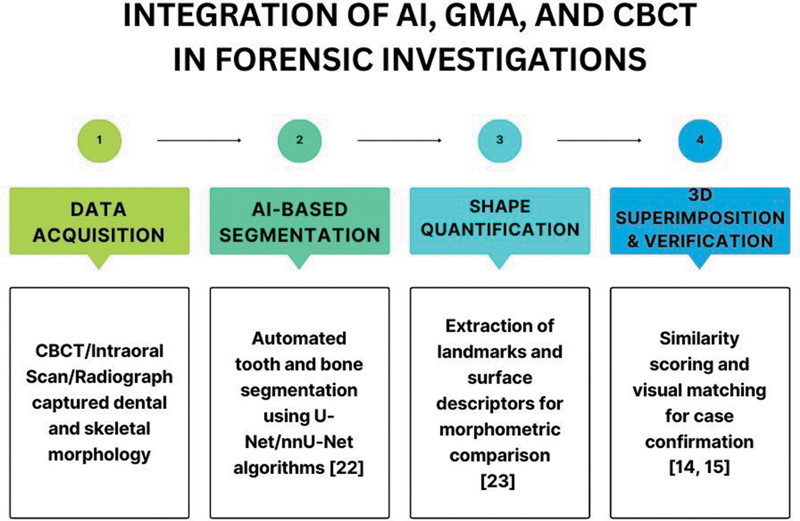
Conceptual workflows illustrating the integration of cone-beam computed tomography (CBCT) imaging, AI-based segmentation, and geometric morphometrics (GMA) in forensic identification pipelines. This process combines automated data extraction, morphometric quantification, and predictive modeling under ethical and legal compliance.

The integration of AI and GMA into forensic workflows provides robust instruments for biological profiling, encompassing sex, age, and identification estimation. Nonetheless, issues such as demographic bias, annotating variability, and the absence of uniform benchmarks must be resolved to guarantee accuracy and equity 38 ( Fig. 2 ). Medico-legal validation, elucidation, and transparent documentation of errors are crucial for judicial admissibility. Through the meticulous incorporation of legal, ethical, and technological safeguards, AI-assisted forensic techniques can achieve scientific rigor and legal defensibility ( Fig. 2 ).
